# Recurrent Peritoneal Pseudocyst: A Rare Complication of Peritoneal Dialysis

**DOI:** 10.7759/cureus.3043

**Published:** 2018-07-24

**Authors:** Savitha Nagaraj, Muhammad H Khan, Farzana Afroza

**Affiliations:** 1 Internal Medicine, The Brooklyn Hospital Center, Brooklyn, USA; 2 Geriatrics/Internal Medicine/The Brooklyn Hospital Center, The Brooklyn Hospital Center, Brooklyn, USA

**Keywords:** spontaneous bacterial perio, peritoneal dialysis, loculated peritoneal pseudocyst, esrd (end stage renal disease), abdominal distension, delayed complication, ascitis

## Abstract

An alarming 468,000 people are dependent on dialysis for their end-stage renal disease (ESRD) management in the United States alone. Peritoneal dialysis is a preferred type of dialysis over hemodialysis, considering its initial survival advantage, patient satisfaction, and cost-effectiveness. One of the rare complications of peritoneal dialysis is abdominal and peritoneal pseudocyst formation. Literature regarding the accurate medical management of such peritoneal pseudocysts is scarce. Adding to this, management of recurrent loculated, non-malignant peritoneal pseudocyst poses to be challenging especially when pseudocysts recur after the offending peritoneal dialysis catheter is removed.

We report one such case of a patient with a history of ESRD managed on long-term peritoneal dialysis. He presented to the hospital with recurrent abdominal pain which was treated multiple times for spontaneous bacterial peritonitis. Due to recurrence, his peritoneal dialysis was discontinued and hemodialysis was initiated. While on hemodialysis and two years after peritoneal dialysis catheter removal, he presented with sudden onset abdominal distension. Imaging showed loculated peritoneal pseudocyst with multiple loculations. Standard recommendation of surgical removal of cyst could not be performed in this patient due to his coexisting medical co-morbidities. Interventional radiology (IR) guided cyst drainage was attempted but was limited due to multiple locutions. However, IR drainage proved to provide temporary relief and after repeated IR guided drainage, a temporary drainage tube was placed. This subsided the recurrence of fluid-filled pseudocysts and the patient improved.

This case emphasizes the importance of follow up of patients who have been or currently are on peritoneal dialysis for early recognition of late-onset complications. Our case also shows the routine challenges faced by the clinician when rare complications arise and standard treatment options cannot be applied.

## Introduction

According to the National Kidney Foundation, 660,000 people are diagnosed with an end-stage renal disease (ESRD) and over 468,000 are on dialysis in the United States alone [1]. A substantial number of these patients are on peritoneal dialysis. Peritoneal dialysis is considered a superior option compared to hemodialysis especially with its increased initial survival advantage, patient satisfaction, and cost-effectiveness. Some of the well-known complications of peritoneal dialysis are leakage, peritonitis, and abdominal wall weakness. However, only a few cases of abdominal and peritoneal pseudocyst formation as a complication of this form of dialysis are reported.

We report one such case of multiple recurrent peritoneal loculated pseudocysts, not responsive to routine management, that occurred as a complication of peritoneal dialysis two years after the peritoneal dialysis was discontinued. This case report will highlight the timeline of the development of the pseudocyst and the challenges in the management of the recurrent pseudocyst in our patient. Our case also emphasizes the importance of long-term follow up of patients on peritoneal dialysis for the development of complications.

## Case presentation

**Figure 1:**
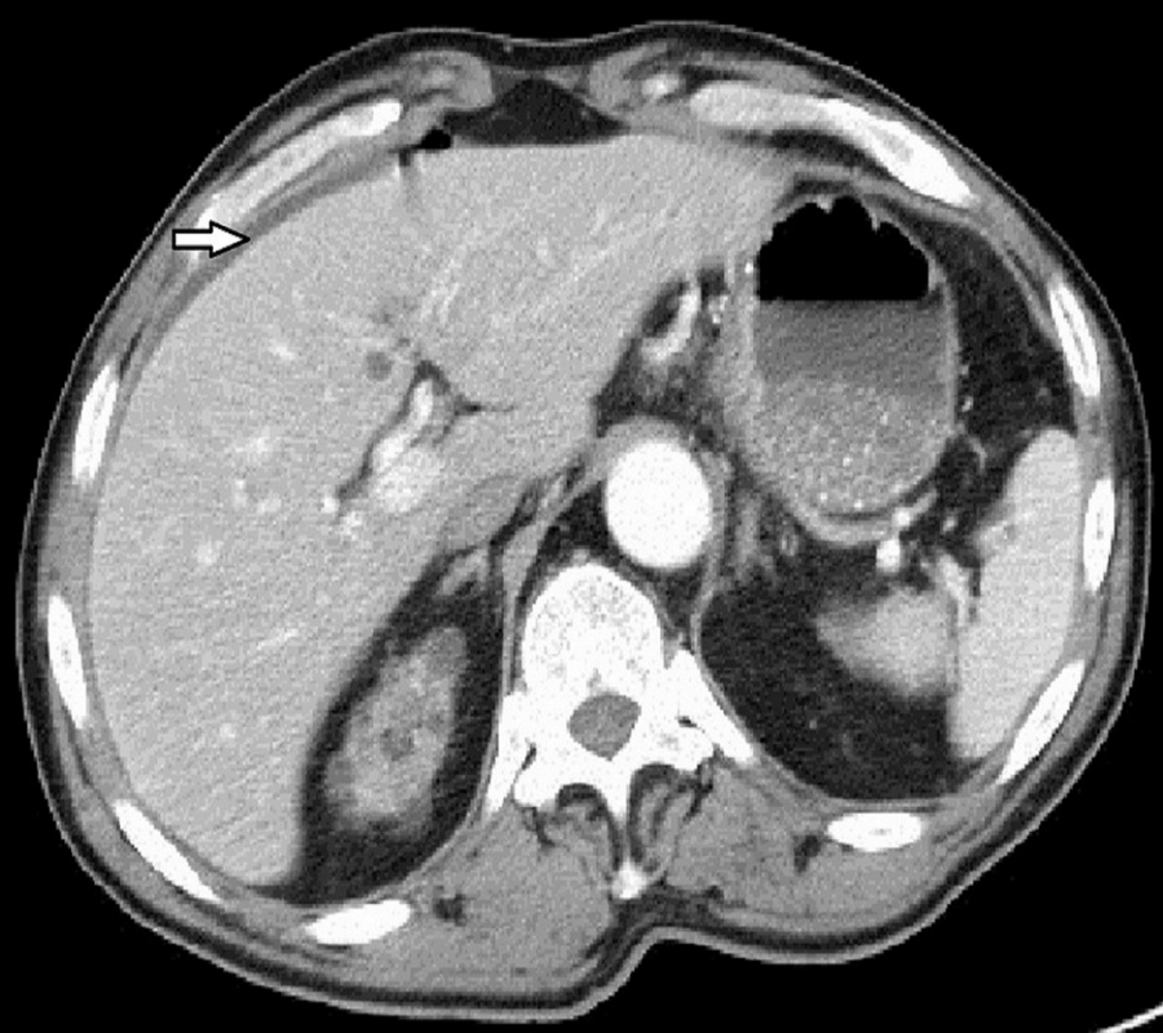
Computed tomography (CT) scan of the abdomen and pelvis showing a small amount of free fluid in the peritoneal cavity

A 68-year-old male with past medical history of ESRD on peritoneal dialysis, hypertension, hyperlipidemia, seizure disorder, left craniectomy was sent from his nursing home in 2013 for abdominal pain, decreased appetite, lethargy, and low oxygen saturation. Examination revealed a cachectic male with tender distended abdomen. Peritoneal dialysis catheter was visualized and the site was clean. He was suspected to have subacute bacterial peritonitis secondary to peritoneal dialysis and was empirically treated with vancomycin and cefepime. Computed tomography (CT) of the abdomen and pelvis showed a small amount of free fluid in the peritoneal cavity (Figure 1).

However, the peritoneal fluid analysis did not grow any organism. Peritoneal dialysis was temporarily discontinued and a temporary hemodialysis catheter was placed. He was later discharged on peritoneal dialysis after his symptoms improved.

The patient presented with similar complaints in 2014 and a single-photon emission computed tomography (SPECT) gallium scan revealed abnormal activity in the right lower quadrant of abdomen and pelvis, suspicious for peritonitis. CT abdomen showed pneumoperitoneum and ascites, peritoneal fluid again did not grow any organisms. Due to elevated leukocyte count and fever, the patient was empirically treated with antibiotics. He was readmitted in 2015 with similar complaints when interventional radiology (IR) guided hemodialysis catheter was placed and peritoneal dialysis was permanently discontinued.

**Figure 2:**
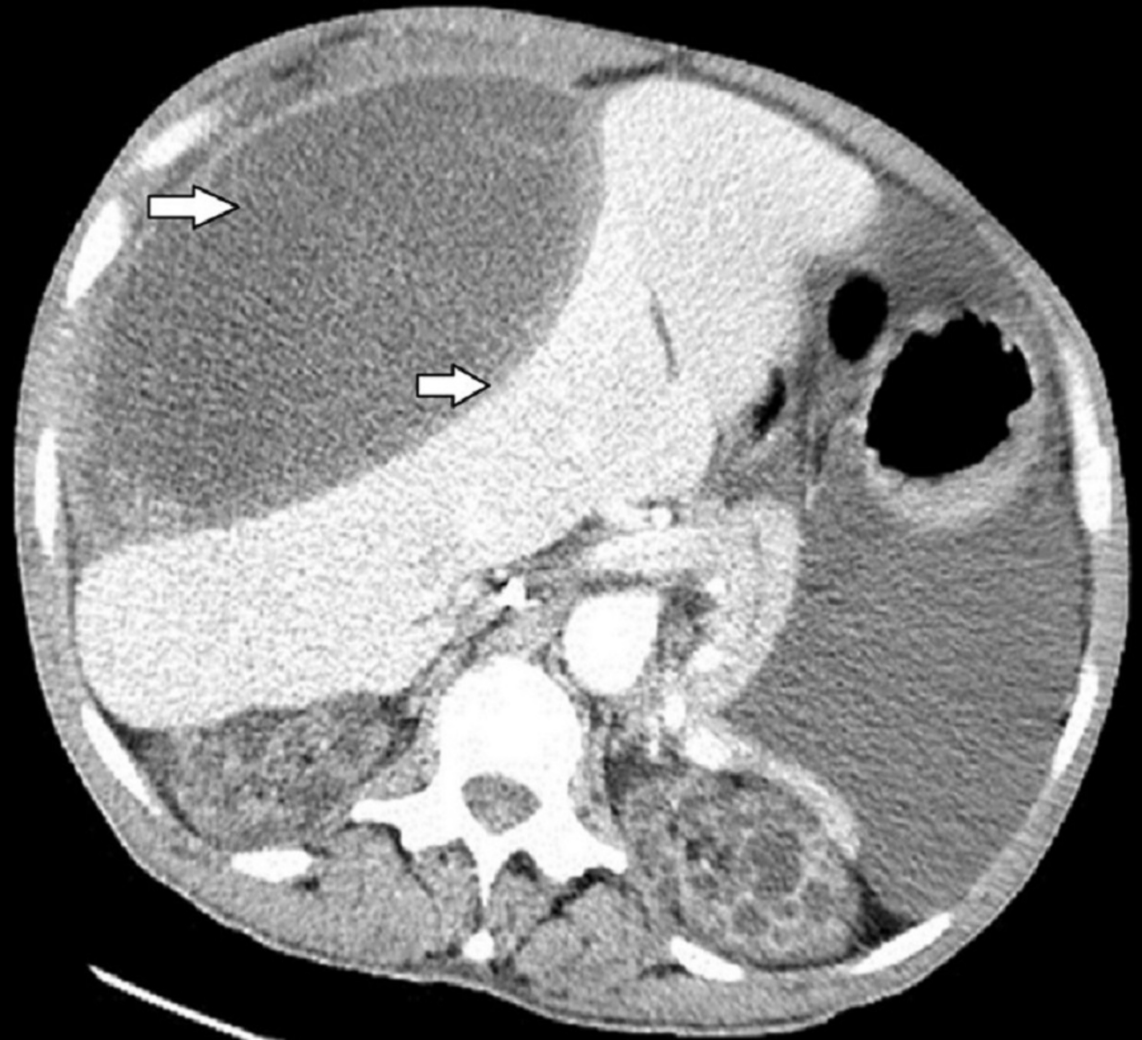
Computed tomography (CT) scan of the abdomen and pelvis revealing encapsulated complex pseudocyst arising from the peritoneal membrane impinging on the liver

The patient presented two years after the discontinuation of peritoneal dialysis with massive abdominal distension, abdominal pain, and vomiting. He was receiving hemodialysis at this time. Repeat CT scan of abdomen and pelvis revealed massive abdominal and pelvic ascites with encapsulated complex pseudocyst arising from the peritoneal membrane impinging on the liver (Figure 2).

**Figure 3:**
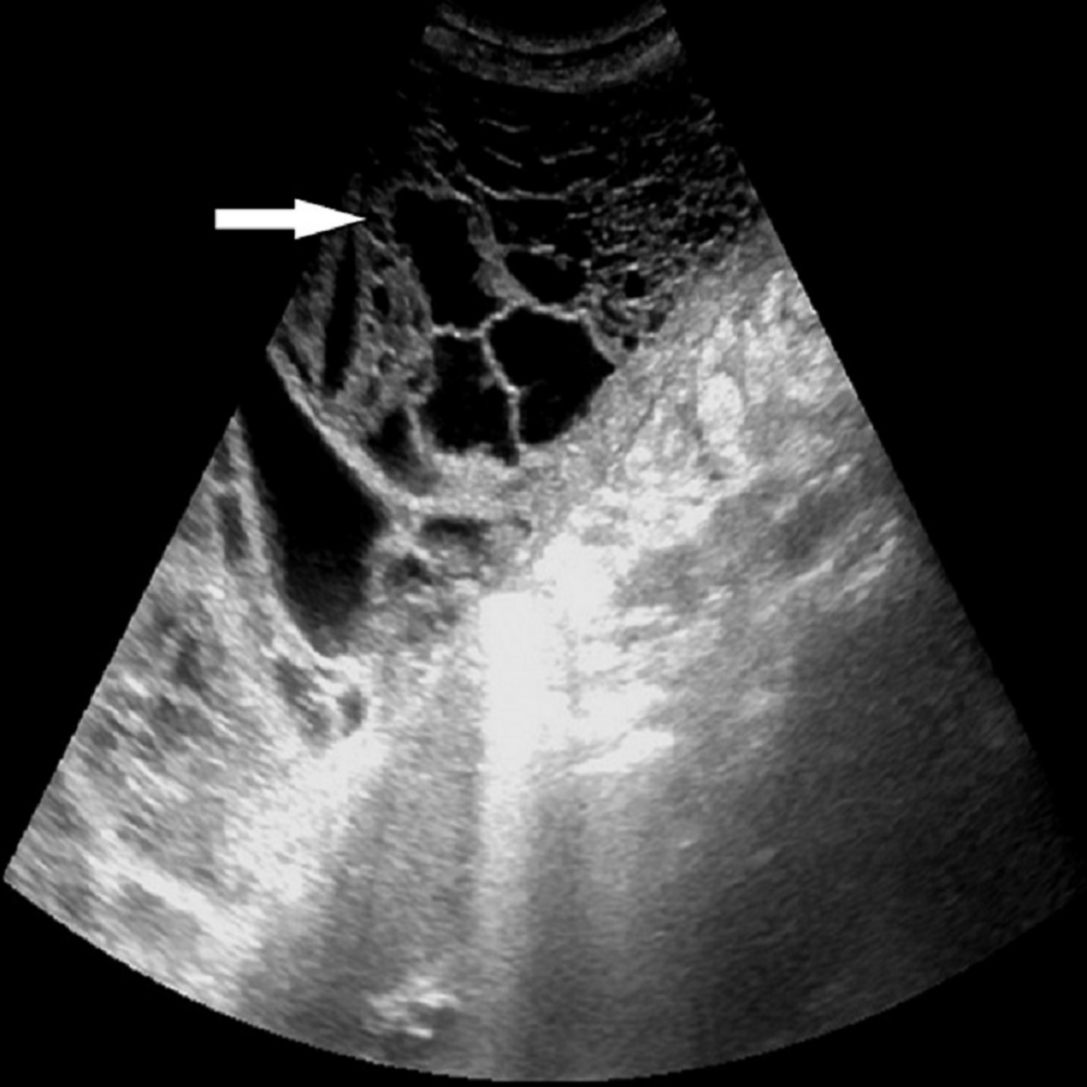
Ultrasound of the abdomen showing multiple fluid-filled loculations

Ultrasound of the abdomen and pelvis showed multiple fluid-filled loculations present within the cyst (Figure 3).

The pseudocysts were suspicious for malignancy; however, IR guided drainage revealed chocolate brown fluid that was negative for malignant cells or organisms.

He presented three more times in the following four months with similar complaints. Surgical management with pseudocyst removal was considered but deferred due to the loculated nature of the cyst and patient’s comorbidities. Medical management with peritoneal drainage drained large amounts of fluids although the procedure was limited by the loculations. The abdominal swelling decreased and the patient experienced temporary relief after every drainage. CT guided subcutaneous peritoneal drainage catheter was temporarily placed and the patient continued to improve. He continues to have occasional episodes of abdominal distension secondary to fluid collection in the pseudocysts although the frequency of recurrence drastically reduced.

## Discussion

Peritoneal dialysis, although a preferred method of dialysis in ESRD patients due to its many advantages, is also associated with a few dangerous complications such as peritonitis and peritoneal pseudocysts. Peritoneal pseudocyst is a less well known but an uncomfortable and dangerous complication of peritoneal dialysis associated with increased risk of recurrent peritonitis. Although there are reported cases of peritoneal pseudocyst as a complication of the ventriculoperitoneal shunt, there are very few reported cases of peritoneal pseudocysts as a complication of peritoneal dialysis [2].

Precise pathophysiology of pseudocyst formation and standardized management guidelines of peritoneal pseudocyst are currently scarce due to the limited number of known cases. In patients with peritoneal dialysis, whether the pseudocyst occurs secondary to chronic irritation of the peritoneum or as a result of recurrent bacterial infection is unknown. Surgical removal of a symptomatic peritoneal pseudocyst is considered curative, but in cases such as our patient, surgical interventions are not an option due to the recurrent infections or other comorbidities that make the patients high risk for surgery. Alternatively draining the cyst via CT guided intervention proved to be a viable option for our patient, although the procedure was limited due to the loculated nature of the cyst.

## Conclusions

Our case report highlights some of the serious, recurrent complications of peritoneal dialysis and the challenges that can be faced in diagnosing and managing these complications. The surprising and puzzling aspect of this case is the time lag between discontinuation of peritoneal dialysis and the development of peritoneal pseudocyst along with the unknown cause and mechanism of the development of pseudocyst. In conclusion, it is important to educate and monitor patients currently or previously on peritoneal dialysis for the development of delayed complications such as pseudocysts and adequately manage these challenging complications. Further studies are needed in this area to look into the pathophysiology and evaluate the contributing factors for the development of peritoneal pseudocyst as a complication of peritoneal dialysis.
